# Dysfunctional cerebello-cerebral network associated with vocal emotion recognition impairments

**DOI:** 10.1093/texcom/tgad002

**Published:** 2023-01-11

**Authors:** Marine Thomasson, Leonardo Ceravolo, Corrado Corradi-Dell’Acqua, Amélie Mantelli, Arnaud Saj, Frédéric Assal, Didier Grandjean, Julie Péron

**Affiliations:** Clinical and Experimental Neuropsychology Laboratory, Department of Psychology, University of Geneva, 40 bd du Pont d’Arve, Geneva 1205, Switzerland; Neuroscience of Emotion and Affective Dynamics Laboratory, Department of Psychology and Swiss Centre for Affective Sciences, University of Geneva, 40 bd du Pont d’Arve, Geneva 1205, Switzerland; Cognitive Neurology Unit, Department of Neurology, University Hospitals of Geneva, Rue Gabrielle-Perret-Gentil 4, Geneva 1205, Switzerland; Neuroscience of Emotion and Affective Dynamics Laboratory, Department of Psychology and Swiss Centre for Affective Sciences, University of Geneva, 40 bd du Pont d’Arve, Geneva 1205, Switzerland; Theory of Pain Laboratory, Department of Psychology, Faculty of Psychology and Educational Sciences (FPSE), University of Geneva, 40 bd du Pont d’Arve, Geneva 1205, Switzerland; Geneva Neuroscience Centre, University of Geneva, Rue Michel-Servet 1, Geneva 1206, Switzerland; Clinical and Experimental Neuropsychology Laboratory, Department of Psychology, University of Geneva, 40 bd du Pont d’Arve, Geneva 1205, Switzerland; Department of Psychology, University of Montreal, Montreal, 90 avenue Vincent d'Indy Montréal, H2V 2S9 Montréal, Québec, Canada; Cognitive Neurology Unit, Department of Neurology, University Hospitals of Geneva, Rue Gabrielle-Perret-Gentil 4, Geneva 1205, Switzerland; Faculty of Medicine, University of Geneva, Rue Michel-Servet 1, Geneva 1206, Switzerland; Neuroscience of Emotion and Affective Dynamics Laboratory, Department of Psychology and Swiss Centre for Affective Sciences, University of Geneva, 40 bd du Pont d’Arve, Geneva 1205, Switzerland; Clinical and Experimental Neuropsychology Laboratory, Department of Psychology, University of Geneva, 40 bd du Pont d’Arve, Geneva 1205, Switzerland; Cognitive Neurology Unit, Department of Neurology, University Hospitals of Geneva, Rue Gabrielle-Perret-Gentil 4, Geneva 1205, Switzerland

**Keywords:** cerebellum, vocal emotion

## Abstract

Vocal emotion recognition, a key determinant to analyzing a speaker’s emotional state, is known to be impaired following cerebellar dysfunctions. Nevertheless, its possible functional integration in the large-scale brain network subtending emotional prosody recognition has yet to be explored. We administered an emotional prosody recognition task to patients with right versus left-hemispheric cerebellar lesions and a group of matched controls. We explored the lesional correlates of vocal emotion recognition in patients through a network-based analysis by combining a neuropsychological approach for lesion mapping with normative brain connectome data. Results revealed impaired recognition among patients for neutral or negative prosody, with poorer sadness recognition performances by patients with right cerebellar lesion. Network-based lesion-symptom mapping revealed that sadness recognition performances were linked to a network connecting the cerebellum with left frontal, temporal, and parietal cortices. Moreover, when focusing solely on a subgroup of patients with right cerebellar damage, sadness recognition performances were associated with a more restricted network connecting the cerebellum to the left parietal lobe. As the left hemisphere is known to be crucial for the processing of short segmental information, these results suggest that a corticocerebellar network operates on a fine temporal scale during vocal emotion decoding.

## Introduction

The study of the functional integration of the cerebellum in the recognition of vocal emotions (i.e. emotional prosody) is a largely neglected topic that deserves to be explored, given the clinical disorders that may result from it. In this context, this study will focus on investigating how deficits in the perception of emotions conveyed by the voice can be linked to cerebellar lesions and what large-scale brain networks could be associated with the latter. The objective of this study was to increase evidence of cerebellar functional involvement in this processing in the literature. Neuroimaging studies in healthy participants have reported the activation of the vermis, posterior cerebellar regions (crus I, lobules VI, VIIb, VIII, and IX), and deep nuclei (dentate and fastigial nuclei) during vocal emotion processing (Imaizumi et al. 1997; Wildgruber et al. 2005; Alba-Ferrara et al. 2011; Kotz et al. 2013; Ceravolo et al. 2021). Moreover, studies on patients with focal cerebellar damage have highlighted the involvement of the cerebrum parvum in the recognition of vocal emotion by identifying associations between deficits in this function (Adamaszek et al. 2014, 2019) and lesions in the right lobules VIIb and VIII and right crus I and II (Thomasson et al. 2019, 2021). Based on these empirical observations, recent theoretical propositions have focused on the “functional specialization” of the cerebellum in emotion processing (Pierce and Péron 2020; Stockert et al. 2021). By generating temporally structured event representations, it is thought that the cerebellum allows for the optimum processing of the salient parameters for a proper emotional event. In cooperation with the cerebellum, the basal ganglia also contribute to the smooth running of this emotion processing by using the cerebellum’s internal model to recruit and synchronize the activity of the relevant cortical and subcortical structures. In the case of emotional prosody processing, neuroimaging studies indicate close cooperation between the cerebellum and all the regions that are recruited at each processing stage, namely, the superior temporal and inferior frontal cortices (Schirmer and Kotz 2006). This leads to the critical—and largely unaddressed—question of the cerebellum’s role in the large-scale brain networks (i.e. “functional integration”) elicited during emotional prosody processing. However, it would appear that the cerebellum interacts with several cortical and subcortical structures known to underlie the 3 different stages of emotional prosody processing developed by Schirmer and Kotz (2006).

The first step of the seminal multistage model of Schirmer and Kotz (2006) consists of the early sensory analysis of relevant perceptual cues (e.g. pitch, loudness, and spectral aspects) and would be subtended by the auditory cortex; the cerebellum communicates indirectly with the auditory cortex via the dorsolateral pontine nuclei (Aitkin and Boyd 1978; Huffman and Henson Jr 1990). More specifically, the right lobules VIIIA and VIIIB and bilateral lobule VI have been identified as being specifically activated for auditory stimulus processing (Baumann and Mattingley 2010). Furthermore, cerebellar lesions have been found to disturb auditory processing by substantially raising thresholds in duration (Ivry and Keele 1989) and pitch discrimination tasks (Parsons et al. 2009). In the same way, a clinical study conducted among 24 patients with focal cerebellar stroke showed that acoustic features, such as fundamental frequency (*F*0), amplitude, and energy distribution, explained a significant proportion of the variance in patients’ vocal emotional misattribution (Thomasson et al. 2021). Critically, however, the cerebellum might not only be involved exclusively in the primary sensory analysis of vocal stimuli but could also participate in the second step described in Schirmer and Kotz (2006)’s model, namely the integration of emotionally meaningful perceptual cues, via connections between the auditory associative areas in the superior temporal gyrus and supratemporal plane and the lateral and dorsolateral pontine nuclei (O'reilly et al. 2010; Baumann et al. 2015). In this perspective, studies have highlighted a contralateral structural connectivity pattern with a loop between the right cerebellum and left temporal cortex (Petersen et al. 1989; Raichle et al. 1994; Riecker et al. 2005, 2006; Ackermann et al. 2007), especially superior temporal sulcus (Stockert et al. 2021). This argues in favor of cerebellar participation in the identification of linguistic auditory objects and thus more broadly in language processing. Finally, the cerebellum is also highly likely to contribute to the third and final stages of emotional prosody processing, reflecting cognitive assessment and emotional meaning. In this step, emotional information is derived from the previous level for higher-order cognitive processes, such as making judgments. This process is thought to be mediated by the right inferior frontal gyrus (IFG) and orbitofrontal cortex, 2 areas that cooperate closely with the cerebellum, notably in the decision-making process (Rosenbloom et al. 2012) and moral judgment (Moll et al. 2001; Greene and Haidt 2002). Moreover, during this final stage, semantic processing recruits the left inferior frontal cortex. Interestingly, robust activation of Broca’s area (left IFG pars opercularis and pars triangularis), as well as the superior medial frontal gyrus and right cerebellum, has been observed in healthy controls (HC) during semantic judgment (Harris et al. 2006). Taken together, these findings indicate that the cerebellum plays a key role in each stage of emotional prosody decoding, particularly through its structural and functional links with the cortical and subcortical structures involved in this processing. However, to our knowledge, no study has clearly investigated how deficits in the recognition of emotions conveyed by the voice can be linked to cerebellar lesions in terms of the impact of brain insults may have on other interconnected brain structures.

In this context, the aim of the present study was to identify the large-scale brain networks associated with deficits in vocal emotion recognition following cerebellar stroke. To this end, we mapped the neural correlates of lesion-induced emotional deficits. More specifically, we first combined lesional information with normative connectome data from typical brains (*n* = 97) matched with our patient sample (cerebellar stroke patients; *n* = 27) and then studied the links between patients’ emotional performances and the brain regions most likely to exhibit dysfunctional responses owing to cerebellar lesions. On the basis of prior findings (Thomasson et al. 2019, 2021, 2022), we expected to observe a greater deficit in the recognition of vocal expressions (for all emotions, but not for neutral) in the patient group than in HC and more particularly in patients with right cerebellar lesions following stroke (RCBL). Moreover, we predicted that the patients’ performance on emotional prosody recognition would be well explainable in terms of functional connections between the damaged site and cortical areas involved in emotional prosody processing—namely bilateral middle and anterior parts of the superior temporal sulcus; inferior and medial frontal regions; and the thalamus, supramarginal gyrus (SMG), amygdala, and basal ganglia (Belin et al. 2000; Ethofer et al. 2012).

## Materials and methods

### Participants

We recruited a sample of 27 patients with first-ever cerebellar ischemic stroke (>3 months prior to enrolment and corresponding to chronic poststroke phase) and 1 group of 27 HC (see Table 1). The data of 24 of the 27 patients had already been acquired in a previous study (Thomasson et al. 2021). The patient sample was divided into 2 groups: 16 patients with RCBL, and 11 patients with left cerebellar lesions (LCBL). The mean age of the RCBL group was 60 years (SD = 11.89, range = 45–85), and the mean age of the LCBL group was 62.4 years (SD = 10.15, range = 43–77). According to the criteria of the Edinburgh Handedness Inventory (Oldfield 1971), 25 patients were right-handed and 2 were left-handed. The mean education level was 16.2 years (SD = 4.54, range = 9–22) for the RCBL group and 12.6 years (SD = 4.10, range = 7–20) for the LCBL group. These 2 groups were matched for sex (*z* = 1.16, *P* = 0.25), age (*z* = 0.89, *P* = 0.37), education level (*z* = −1.82, *P* = 0.07), and handedness (*z* = 0.22, *P* = 0.83). All patients were French speakers. Mean time since stroke was 26.7 months (SD = 32.93, range = 3–155). Exclusion criteria were (i) brainstem or occipital lesion (factor known to influence clinical signs), (ii) at least one other brain lesion, (iii) diffuse and extensive white-matter disease, (iv) other degenerative or inflammatory brain disease, (v) confusion or dementia, (vi) major psychiatric disease, (vii) the wearing of hearing aids or a history of tinnitus, or a hearing impairment as attested by the Montreal Toulouse auditory agnosia battery (PEGA; Agniel et al. 1992) (mean total score = 28.4, SD = 1.9, range = 24–30), (viii) age <18 years, and (ix) major language comprehension deficits precluding reliable testing. All the tasks described below were designed to be highly feasible even for patients in clinical settings.

**Table 1:** Clinical, demographic, and neuropsychological data of the 2 subgroups of patients with cerebellar stroke.

Patient	Age (years)	Sex	Handedness	Education (years)	Side of lesion	SARA	MOCA	FAB	Cat. fluency	Act. fluency	BDI-II	TAS-20	AES	Lesion volume (voxels)	Lesion location
P1	52	M	Right	20	RH	0	21^a^	12^a^	6^a^	3^a^	23^a^	45	1	5,546	Lobules IV, V, VI, VIII and crus I
P2	55	M	Right	22	RH	–	23^a^	–	16^a^	11	5	–	–	3,566	Lobules III, IV, V, VI, VIII, crus I and Vermis III
P3	56	F	Right	15	LH	–	18^a^	–	31	14	17	–	–	652	Lobules VIII, VIIb and Vermis VIII
P4	62	F	Left	12	LH	7.5	30	13^a^	25	19	15	36	7	22,038	Lobules III, IV, V, VI, VIIb, VIII, IX, crus I, II and Vermis I, II, VI, VIII, IX, X
P5	73	F	Right	18	RH	–	19^a^	–	15^a^	5^a^	24^a^	–	–	25	Crus I
P6	61	F	Left	9	RH	3	23^a^	13^a^	16^a^	9	23^a^	63^a^	10	6,221	Lobules VIIb, VIII, IX, crus II and Vermis VIII
P7	58	M	Right	20	LH	0	30	17	19^a^	17	5	72^a^	0	15,103	Lobules VIIb, VIII, IX, crus I, II and Vermis IX
P8	68	F	Right	14	LH	3.5	24^a^	14^a^	5^a^	16	19^a^	61^a^	0	3,309	Lobules VI, VIIb, VIII, IX and crus I, II
P9	50	M	Right	19	RH	0	26	15^a^	18^a^	19	5	36	0	53	Lobule VIII and Vermis VIII
P10	50	F	Right	19	RH	0	27	18	20^a^	29	–	–	0	1,254	Lobules IV, V, VI and crus I, II
P11	77	M	Right	9	LH	6.5	22^a^	12^a^	10^a^	11	7	35	6	1,605	Lobules VI, VIIb, VIII, IX, crus I and Vermis IX
P12	72	M	Right	20	RH	6	28	15^a^	18	20	11	46	2	19	Lobule VIII
P13	43	M	Right	10	LH	2	22^a^	9^a^	16^a^	14	19	69	13	6,396	Lobule VIIb, VIII and crus I, II
P14	73	M	Right	10	RH	2	23^a^	17	10^a^	4^a^	17	84	1	35	Lobule VI and crus I
P15	74	F	Right	13	RH	1	26	18	19	13	8	61^a^	0	38,489	Lobules VI, VIIb, VIII, IX, X, crus I, II and Vermis IV, V, VI, VII, VIII, IX, X
P16	66	F	Right	19	LH	1	27	16	27	22	2	40	0	351	Lobules IV, V, VI, VIII
P17	53	M	Right	19	RH	0	26	17	31	21	12	45	1	23,290	Lobules VI, VIIb, VIII, XI, X and crus I, II
P18	85	M	Right	12	RH	5	19^a^	11^a^	14	7	10	66^a^	2	239	Lobules VIIb, VIII and crus I, II
P19	76	F	Right	9	LH	1	24^a^	14^a^	18	14	10	52	0	13	Lobules IV, V, VI
P20	69	M	Right	7	LH	0	27	14^a^	15	7	5	34	9	31	Lobule VIII
P21	52	M	Right	9	RH	0	25^a^	13^a^	9^a^	7	10	43	0	692	Lobules VIIb, VIII and crus II
P22	58	M	Right	12	LH	0.5	27	16	14^a^	9	21^a^	84^a^	19^a^	4,206	Lobules III, IV, V, VI and crus I
P23	48	F	Right	15	RH	0	25^a^	18	33	22	14	34	0	3,064	Lobules VI, VIIb, VIII and crus I, II
P24	53	F	Right	12	LH	0	22^a^	17	20	16	10	59	3	6,520	Lobules VIIb, VIII, IX and crus I, II
P25	54	M	Right	20	RH	0	27	18	35	–	7	52	0	3,829	Lobules VIIb, VIII and crus II
P26	45	M	Right	20	RH	0	29	18	29	39	16	56	0	15,439	Lobule VI and crus I, II
P27	63	M	Right	12	RH	0	26	16	27	12	3	51	0	4,840	Lobules VIII, IX

Note. Dashes indicate missing values. M, male; F, female; RH, right hemisphere; LH, left hemisphere; Act. fluency, action verb fluency task; AES, Apathy Evaluation Scale; BDI-II, Beck Depression Inventory; Cat. fluency, categorical fluency task; FAB, Frontal Assessment Battery; MOCA, Montreal Cognitive Assessment; PEGA, Montreal-Toulouse auditory agnosia battery; SARA, Scale for the Assessment and Rating of Ataxia; TAS-20, Toronto Alexithymia Scale.

^a^Scores below the clinical threshold.

The participants in the HC group had no history of neurological disorders, head trauma, anoxia, stroke, or major cognitive deterioration, as attested by their score on either the Mattis Dementia Rating Scale (Mattis 1988) (mean score = 142.2, SD = 1.7, range = 140–144), the French version of the modified Telephone Interview for Cognitive Status (Lacoste and Trivalle 2009) (mean score = 34.9, SD = 4.6, range = 32–40), or the Montreal Cognitive Assessment (Nasreddine and Patel 2016) (mean score = 27.8, SD = 1.5, range = 26–30). They were all French speakers with a mean age of 60.8 years (SE = 10.43, range = 40–80). According to the Edinburgh Handedness Inventory criteria (Oldfield 1971), 25 participants were right-handed and 2 were left-handed. Their mean education level was 14.96 years (SE = 3.7, range = 9–22). As with the patient sample, none of the HC wore hearing aids or had a history of tinnitus, or a hearing impairment as attested either by their PEGA score (mean = 28.15, SD = 2.2, range = 26–30) or by the results of a standard audiometric screening procedure (AT-II-B audiometric test) to measure the tonal and vocal sensitivities.

All participants gave their written informed consent, and the study was approved by the local ethics committee.

### Experimental protocol

#### Neuropsychological and psychiatric data

First, all patients performed a motor scale to quantify their cerebellar ataxia (Scale for the Assessment and Rating of Ataxia; Schmitz-Hubsch et al. 2006). We then administered a set of neuropsychological tests that included the Montreal Cognitive Assessment (Nasreddine and Patel 2016) and a series of tests assessing executive functions: the Frontal Assessment Battery (Dubois et al. 2000), categorical and literal fluency tasks (Cardebat et al. 1990), and an action verb fluency task (Woods et al. 2005).

Participants also completed further psychiatric questionnaires at home, assessing depression (Beck Depression Inventory [BDI]) (Steer et al. 2001) and alexithymia (Toronto Alexithymia Scale; Bagby et al. 1994). Moreover, as apathy symptoms are commonly found in patients with cerebellar stroke (Villanueva 2012), we administered the Apathy Evaluation Scale (Marin et al. 1991).

Finally, participants performed the emotional prosody recognition task. The entire protocol was completed within a single session lasting approximately 90 min.

#### Vocal emotion recognition task procedure

This emotional prosody recognition task was composed of 60 pseudowords pronounced by 12 different actors (6 women and 6 men) each in 1 of 5 different prosodies (anger, fear, happiness, neutral, and sadness).

After listening to a stimulus played bilaterally through stereo headphones, participants were instructed to rate its emotional content on a set of scales displayed simultaneously on the computer screen. This implied indicating the extent to which a voice expressed different emotions by moving a cursor along a visual analog scale ranging from “No emotion expressed” to “Emotion expressed with exceptional intensity.” Six scales were displayed: 1 scale for each emotion played (anger, happiness, fear, and sadness) and 1 for neutral utterances. Additionally, we also included a scale to rate the surprise emotion in order to find out whether participants confused the fear emotion expressed by the human voice with surprise, which can be the case with facial and vocal expressions (Banse and Scherer 1996; Ekman 2003; Scherer and Ellgring 2007).

#### Standard protocol approvals, registration, and patient consent

Written informed consent was obtained from each participant, the study met the ethical standards of the responsible committee on human experimentation, and was conducted in accordance with the Declaration of Helsinki.

#### Lesion mapping

The brain images were acquired in a 1.5T MRI scanner when patients were admitted to hospital. The mean time between stroke and image acquisition was 1.67 days (SE = 1.90, range = 0–7). All the lesions were mapped on diffusion-weighted (25 patients) or CT (2 patients) brain scans using the Clusterize-toolbox (http://www.medezin.uni-tuebingen.de/kinder/en/research/neuroimaging/software/). This method consists of the automated identification of local lesion clusters on each image slice based on its intensity, followed by manual validation and potential freehand correction (Clas et al. 2012; De Haan et al. 2015). The resulting lesion map was then normalized to the Montreal Neurological Institute (MNI) single-subject template, with a resolution of 1 × 1 × 1 mm voxel size using SPM12 software (http://www.fil.ion.ucl.ac.uk/spm/). In particular, we applied a deformation field estimated from a T2 (*n* = 25) or CT (*n* = 2) brain scan registered to each map.

### Statistical analysis

#### Behavioral data

##### Sociodemographic and clinical data

As sociodemographic and clinical variables were not normally distributed, comparisons between the 3 groups (LCBL, RCBL, and HC) were performed using Kruskal-Wallis tests. Mann–Whitney tests were also used when a comparison between 2 independent groups was necessary (RCBL vs. LCBL). As the data for the age variable followed a normal distribution, we performed single-factor analysis of variance. If the latter yielded a significant difference, we ran pairwise *t*-tests for 2 independent groups to determine which groups differed from one another. False discovery rate (FDR) correction for multiple comparisons was applied.

##### Vocal emotion recognition data

Previous studies investigating emotional prosody recognition in cerebellar patients had shown that they correctly identify the target emotion but made misattributions regarding nontarget emotions (Thomasson et al. 2019, 2021). We, therefore, calculated a discrimination index reflecting the difference between the rating on the target emotion scale and the averaged ratings on the 5 incorrect emotion scales (i.e. target emotion recognition over nontarget emotions) (Cristinzio et al. 2010). This index was particularly useful for studying the emotion recognition accuracy and yielded information about possible confusions or emotional misattributions for each emotional prosody presented in the task. We calculated a linear mixed model with emotion (5 levels) as the within-participants variable, group (HC, RCBL, and LCBL) as the between-participants variable and participant as the random intercept. We then ran contrasts between the groups for each prosodic category. Each *P* value yielded by the contrasts was corrected for multiple comparisons with the Bonferroni method by dividing the *P* value by the between-groups comparison for each emotion category (0.05/(3 × 5)).

##### Relationship between clinical characteristics and vocal emotion recognition

Moreover, we looked for correlations between the clinical and emotional data for the patient group using Spearman’s rank test, as the distribution of the data was not normal. To avoid type I errors, we only included emotional variables that differed significantly in the analyses, either between patients and HC, or between the 2 patient subgroups (LCBL and RCBL). If significant correlations were found, we calculated the Akaike information criterion (AIC) and Bayesian information criterion (BIC) to see whether the models containing the clinical variables correlating with our emotional data had a better fit than the model that did not contain them. The lower the AIC or BIC value, the better the fit would be.

#### Functional and lesion-based neuroimaging

To identify brain regions that were functionally connected to the damaged cerebellar areas causing the vocal emotion recognition deficits, we combined the standard lesion-symptom mapping with normative functional connectivity data to achieve network-based lesion-symptom mapping (NLSM) (Boes et al. 2015; Laganiere et al. 2016; Darby et al. 2017; Wawrzyniak et al. 2018; Corradi-Dell’Acqua et al. 2020). To do so, we performed a 2-step NLSM analysis: (i) First, we used each of the masks created during the lesion mapping step described above (see the Experimental protocol section) as a seed region of interest (ROI) in a resting-state functional connectivity analysis that used normative connectome data. The latter data were extracted from the OpenfMRI (https://openfmri.org/) database (accession no. ds000221); (ii) second, the resulting network masks were modeled against the patients’ discrimination index calculated for each emotional prosody. These steps are described in details in the following paragraphs.

##### OpenfMRI data selection and extraction

Within this cohort, we selected the data of 97 neurotypical individuals (mean age = 63.17 years, 46 F/51 M) so that they would be matched for age, *t*(122) = 0.93, *P* = 0.86, and sex, *t*(122) = 0.61, *P* = 0.54, with our patient sample. For each participant selected for the present study, from this database, between 1 and 5 resting-state sessions lasting 15 min each were acquired in a 3T Verio whole-body MRI scanner (Siemens, Tarrytown, NY, United States). Functional images were acquired using a 64-channel head-and-neck coil and a multiband imaging sequence with time to recovery = 1,400 ms, time to echo = 39 ms, flip angle = 69°, 64 interleaved slices, 88 × 88 in-plane resolution, 2.3 × 2.3 × 2.3 mm voxel size, and no interslice gap. The multiband acceleration factor was 4. For some participants, several resting-state sessions were available. Consequently, we randomly selected 1 resting-state session for each participant. After the preprocessing/denoising of functional data (see below), we visually inspected each session for potential artifacts in the signal, including global effects, high movements, or presence of artefactual scans. Had we found artifacts, we would have selected another resting-state session for the participant in question.

##### OpenfMRI data preprocessing

Resting-state data were analyzed using a combination of SPM12 (https://www.fil.ion.ucl.ac.uk/spm) and CONN version 20.b (Whitfield-Gabrieli and Nieto-Castanon 2012) preprocessing pipelines for optimum data preprocessing and denoising. More specifically, functional data were realigned to the first volume of the time series to account for head motion, slice-time corrected, and assessed for potential artifacts (using ART toolbox embedded in CONN 20.b https://www.nitrc.org/projects/artifact_detect). Subsequently, data were denoised through the default pipeline in CONN toolbox to remove components in the neural signal which were related to (i) white matter and cerebrospinal fluid signal (first 15 principal components), (ii) estimated subject movement parameters (from preprocessing), and (iii) the presence of outlier scans (estimated through the ART toolbox). Data were also band-pass filtered (0.008–0.09 Hz) to account both for slow-frequency fluctuations (such as scanner drift) as well as physiological and residual movement artifacts. Data were finally normalized to the MNI template (with a 2 × 2 × 2 mm voxel size) and were smoothed by convolution with an isotropic 8-mm full width at half-maximum Gaussian kernel.

##### Functional connectivity analysis

Preprocessed resting-state data were then fed to a seed-based functional connectivity analyses, as implemented in CONN. To this end, we entered ROIs specific to the binary lesion mask of each of the 27 patients in the present study. Hence, for each of 97 individuals from the resting-state cohort, we calculated correlation maps using bivariate Pearson’s correlation coefficients between the average time courses from each of the 27 ROIs and each remaining voxel of the entire brain (ROI-to-voxel analyses). This led to 2,619 (97 × 27) whole brain linear models, each leading to a correlation map that was then converted to normally distributed values using the Fisher transform. Finally, we ran group-level analyses using these Fisher-transformed correlation maps. Type I errors were controlled for by using a 2-tailed FDR correction with *P* < 0.05 to correct for multiple comparisons. These analyses resulted in 27 functional connectivity network maps representing positive and negative linear relations between each ROI (i.e. functional maps showing each lesion of our 27 patients as an individual seed region) and the rest of the brain averaged across our 97 neurotypical participants. However, we retained only positively coupled ROIs to make the interpretation of the link between cerebellar lesions and behavior clearer. Each group-level functional network map (*n* = 27) was then used as input in the NLSM analyses (see next section).

##### NLSM analysis

NLSM analysis consisted of associating network maps of the patients’ lesions with normative connectome data from a matched population to provide an estimate of the brain regions that were functionally connected to the lesion site and which might therefore exhibit dysfunctional properties. In these analyses, each lesion-based functional connectivity network map (*n* = 27) was modeled against the patients’ discrimination index for each emotional prosody. The analysis was restricted to voxels implicated in at least 5% of patients corresponding to a search area of 65,906 voxels (i.e. 527,248 mm^3^). For each voxel, the discrimination index for each type of emotional prosody was fitted against lesion presence using a linear model. To account for potential confounds unrelated to vocal emotion recognition, the linear regression included potential nuisance variables identified in previous behavioral analyses: educational level, time since stroke, and lesion volume. We used permutations to correct the NLSMs for multiple comparisons at the cluster level (*P* < 0.05 familywise, with an underlying height threshold corresponding to *P* < 0.001 uncorrected). Permutations were used to randomly reassign the patients’ behavioral scores 5,000 times. For each permutation, the general linear model was refitted across all search voxels (*n* = 65,906), and the largest cluster was selected. Only the top 5% of the permuted distribution across all voxels in the largest cluster was tagged significant in the original unpermuted data. This method ensured that the probability of such a lesion would be <5% if there was no linear relation between brain and behavior (Nichols and Holmes 2002). This approach suited the nature of our data, namely network masks, including both lesions and resting-state data. This type of analysis technique has been successfully used in previous studies investigating social cognition abilities (e.g. Corradi-Dell'Acqua et al. 2011, 2014, 2016, 2020; Qiao-Tasserit et al. 2018) and in the lesion literature (Pillay et al. 2014, 2017; Mirman et al. 2015; Binder et al. 2016). The analysis was carried out using the latest VLSM package (https://aphasialab.org/vlsm) for MATLAB R2021a (The Mathworks, Natick, MA) software.

## Results

### Sociodemographic and clinical data

Analysis failed to reveal any significant difference between the 2 cerebellar patient subgroups or HC on any of the sociodemographic or clinical data variables using FDR (*P* > 0.05). The 3 groups were comparable for age, *F*(2, 51) = 0.16, *P* = 0.85, education level (χ^2^ = 4.94, *P* = 0.08), handedness (χ^2^ = 0.75, *P* = 0.96), and sex (χ^2^ = 2.55, *P* = 0.28) (Table 2).

**Table 2:** Statistical results of group (LCBL, RCBL, and HC) comparisons on clinical, demographic, and neuropsychological data.

		LCBL subgroup (*n* = 11)		RCBL subgroup (*n* = 16)		HC (*n* = 27)		Stat val.	*P* value
		Mean	±SD	Mean	±SD	Mean	±SD		
Age in years		62.36	10.15	60.00	11.89	60.78	10.43	0.18	0.85
Education level in years		12.64	4.10	16.25	4.54	14.96	3.70	4.94	0.08
MOCA		24.82	3.74	24.56	2.99			0.17	0.86
Verbal fluency	Categorical	18.18	7.52	19.75	8.80			−0.35	0.73
	action	14.45	4.32	14.73	10.19			0.47	0.64
FAB		14.20	2.48	15.92	2.36			−1.71	0.10
AES		5.70	6.50	1.23	2.74			1.52	0.13
PEGA		28.20	2.20	28.61	1.71			−0.22	0.83
SARA		2.20	2.76	1.31	2.10			1.12	0.26
BDI-II		11.82	6.66	12.53	6.84			−0.26	0.79
TAS-20		54.20	17.66	53.08	14.05			0.07	0.95

Note. RCBL, patients with right cerebellar lesions; LCBL, patients with left cerebellar lesions; Stat. val., statistical value; MOCA, Montreal Cognitive Assessment; FAB, Frontal Assessment Battery; AES, Apathy Evaluation Scale; PEGA, Montreal-Toulouse auditory agnosia battery; SARA, Scale for the Assessment and Rating of Ataxia; BDI, Beck Depression Inventory; TAS-20, Toronto Alexithymia Scale.

### Vocal emotion recognition

Overall, the analysis revealed a main effect of emotion, *F*(4, 3174) = 19.57, *P* < 0.001, and an effect of group that tended toward significance, *F*(2, 44) = 2.82, *P* = 0.07. The 2-way interaction between group and emotion was not significant, *F*(8, 3174) = 1.56, *P* = 0.13.

We performed contrasts for each vocal emotion, with Bonferroni correction for multiple comparisons, and obtained the following results:

For “anger,” we found a difference between patients (LCBL and RCBL) and HC that tended toward significance, χ^2^(1) = 3.72, *P* = 0.054, especially between LCBL and HC, χ^2^(1) = 3.79, *P* = 0.052.

For “neutral,” the contrast revealed a significant difference between patients (LCBL and RCBL) and HC, χ^2^(1) = 9.12, *P* = 0.0002. More particularly, we found significant differences between LCBL and HC, χ^2^(1) = 7.27, *P* = 0.007 and between RCBL and HC, χ^2^(1) = 4.97, *P* = 0.026.

**Fig. 1:**
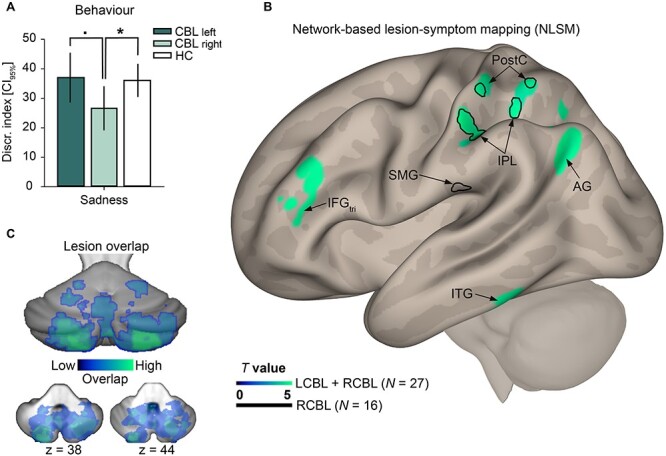
A) Mean discrimination index for sadness prosody for HC, RCBL, and LCBL groups. B) NLSM results. Surface rendering indicates areas with dysfunctional connectivity with cerebellar lesions linked to performances on sadness recognition (discrimination index) performances. Areas from blue to green constitute the network revealed by the NLSM analysis conducted with the maps of all the patients (LCBL and RCBL: *n* = 27). The areas outlined in black correspond to the network yielded by the NLSM analysis performed solely with the maps of patients with a right cerebellar lesion (RCBL; *n* = 16). C) Overlap map showing all the cerebellar lesions exhibited by patients (*n* = 27), ranging from blue (few patients) to green (more patients). CBL, cerebellar; IFGtri: IFG pars triangularis.

For “sadness,” no significant differences were highlighted between patients (LCBL and RCBL) and HC, χ^2^(1) = 1.38, *P* = 0.24, but there was a significant difference between RCBL and HC, χ^2^(1) = 4.71, *P* = 0.03, as well as a difference between LCBL and RCBL that tended toward significance, χ^2^(1) = 3.80, *P* = 0.051 (Fig. 1A).

There were no significant group effects for either “happiness” or “fear” discrimination index (all *P*s > 0.05). See Supplementary Table S1 and Supplementary Fig. S1.

Overall, our data confirm previous studies (Thomasson et al. 2019; Thomasson et al. 2021) by revealing that patients with cerebellar lesion have lower discrimination index with respect to HC, reflecting stronger misattributions for nontarget emotions. This effect changes also as a function of the emotional state addressed, with states like anger/neutral being affected in both clinical groups equally, whereas states like sadness being specifically impaired following right hemisphere lesions, and others showing instead no impairment.

### Relationship between clinical characteristics and vocal emotion recognition

A significant and positive correlation was observed between the “neutral” discrimination index and categorical fluency scores (*r*^Sp^ = 0.48, *P* = 0.03). Moreover, significant negative correlations were revealed between the “neutral” discrimination index and the BDI (*r*^Sp^ = −0.54, *P* = 0.004), Apathy Evaluation Scale (*r*^Sp^ = −0.49, *P* = 0.02), and Scale for the Assessment and Rating of Ataxia (*r*^Sp^ = −0.46, *P* = 0.03) scores. Finally, significant positive correlations were found between the “sadness” discrimination index and the action fluency (*r*^Sp^ = 0.49, *P* = 0.01) and total PEGA (*r*^Sp^ = 0.55, *P* = 0.006) scores. All other correlations were nonsignificant (all *P*s > 0.05). We calculated the AIC and BIC to see whether the models containing the previous clinical variables that correlated with our emotional data had a better fit than the model that did not contain them. Without the action fluency score variable, the AIC was 15,763 and the BIC was 15,849. With the action fluency score variable, the AIC was 15,763 and the BIC was 15,854. Thus, although action fluency scores did correlate with some measures, the overall performance of our sample was better accounted by a model that did not included this score (difference between the 2 models: χ^2^(1) = 3.91, *P* = 0.048). Furthermore, all models, including the other clinical variables (categorical fluency, depression, apathy, ataxia, and auditory agnosia scores), did not differ significantly from the model that did not contain these variables (all *P*s > 0.05), showing no real advantage in the inclusion of these scores in subsequent analytical steps.

### NLSM analysis

The regions significantly highlighted by the NLSM analysis are reported in Table 3 and Fig. 1B and C. More specifically, we found a network involving left frontal (IFG pars triangularis), temporal (inferior temporal gyrus [ITG]), and left parietal (inferior parietal lobule [IPL], postcentral gyrus [postC], and angular gyrus [AG]) regions that correlated with sadness discrimination index. As previous analysis suggested that sadness recognition is particularly impaired in RCBL, we performed another NLSM analysis solely with maps of RCBL patients (*n* = 16). Results highlighted a network composed of regions located solely in the left parietal lobe, more specifically in the left IPL, left postC, and left SMG (Table 3 and Fig. 1B). No other links were found to any other emotion discrimination index.

**Table 3:** NLSM regions and MNI coordinates (5,000 permutations).

Region	Hemisphere	*X*	*Y*	*Z*	*T* statistic	Cluster size
All patients (RCBL + LCBL; *n* = 27)						
Inferior parietal lobule	L	−50	−26	30	4.82	329
postC		−50	−32	58	4.01	
Inferior temporal gyrus	L	−54	−52	−24	4.61	125
Angular gyrus	L	−44	−64	34	4.20	112
Inferior frontal gyrus pars triangularis	L	−40	42	6	3.89	91
Patients with right cerebellar lesion only (RCBL; *n* = 16)						
Inferior parietal lobule	L	−52	−32	42	4.83	125
postC		−52	−34	56	4.26	
Supramarginal gyrus	L	−52	−26	22	4.38	29

Note. NLSM, Network-based lesion-symptom mapping; MNI, Montreal Neurological Institute; postC, postcentral gyrus. *P* < 0.001 corrected.

## Discussion

The aim of the present study was to investigate the lesional correlates underlying emotional prosody recognition in cerebellar stroke patients, by combining a neuropsychological approach with lesion mapping and normative brain connectome data, taking the hemispheric lateralization of the lesions into account. There is increasing neuroimaging and clinical evidence in favor of the functional specialization of the cerebellum during vocal emotion recognition (Adamaszek et al. 2014, 2019; Thomasson et al. 2019, 2021; Ceravolo et al. 2021), but its functional integration in this process deserves more attention. Using NLSM, the present study found that sadness recognition deficits following cerebellar stroke were related to a fronto-temporo-parietal network.

Regarding behavioral results, patients with cerebellar stroke had difficulty recognizing emotional prosody, and deficits were specifically found for negative prosody. These findings are consistent with previous neuroimaging and neurostimulation studies reporting preferential involvement of the cerebellum when processing negative emotional stimuli (Ferrucci et al. 2012; Schraa-Tam et al. 2012; for a review, see Leggio and Olivito 2018). More specifically, RCBL patients did not recognize sadness as well as HC did (with a trend toward a difference from LCBL patients). Cerebellar involvement in sadness processing has also been demonstrated in both neuroimaging (Lane et al. 1997; Liotti et al. 2000; Habel et al. 2005; Vytal and Hamann 2010; Baumann and Mattingley 2012) and clinical (Krüger et al. 2003; Ruggiero et al. 2021) studies. Using NLSM analysis, we found a network involving left frontal (IFG pars triangularis), temporal (ITG), and parietal (IPL and postcentral gyrus and AG) regions that correlated with the sadness discrimination index. In line with our assumptions, some of these areas have previously been described as involved in the processing of emotional prosody. The role of the bilateral IFG has been reported in complex perceptual decision-making (Binder et al. 2004), and more specifically, the left IFG, in the explicit decoding of emotional prosody (Bach et al. 2008; Ethofer et al. 2009; Frühholz et al. 2012) as well as its key role in processing prosodic information used for sentence comprehension (Schirmer and Kotz 2006; van der Burght et al. 2019). In particular, our results corroborate previous studies showing the involvement of the IFG pars triangularis during explicit evaluations of vocal emotions and notably more for voices than faces (Dricu and Frühholz 2016). In the present study, we also found that the sadness discrimination index of patients was associated with the functioning of several brain regions located in the parietal lobe. Interestingly, it seems that the frontoparietal network is overrepresented 2.3-fold in the cerebellum compared to the cortex, occupying more cerebellar volume than any other resting-state network (Marek et al. 2018). A disruption of this network, known to regulate the integration of other association and motor networks (Dosenbach et al. 2007), could indeed be related to a disruption of the low- and high-level information integration processes necessary for the processing of emotional prosody (Schirmer and Kotz 2006). The association between the sadness discrimination index of patients and the functioning of the left AG also seems to be an interesting avenue to explore. Previous studies have shown that anodal stimulation over this structure results in faster comprehension of semantically meaningful combinations (Graves et al. 2010), while another recent study showed that right cerebellar transcranial magnetic stimulation interferes with accuracy in judging the relatedness of meaningful word pairs (Gatti et al. 2020). These differential effects of brain stimulation (i.e. response latency facilitation vs. accuracy impairment) may reflect the different types of semantic integration in cortical areas and in the cerebellum and highlight possible hemispheric crossspecialization between cortical and cerebellar areas.

In this respect, our study interestingly showed that, compared with HC (and, to a lesser extent, LCBL), RCBL patients performed more poorly for the sadness discrimination index and indicated that the ability to recognize this emotion may be linked to the functioning of a neural network that includes the cortical areas located in the left hemisphere. While, there is a large corpus of neurological studies among patients with brain damage, suggesting that the right hemisphere plays an important role in emotional prosody processing (Ross 1981; Tompkins and Flowers 1985; Blonder et al. 1991; Grandjean et al. 2008; Witteman et al. 2011), there is little evidence of similar hemispheric specialization in the cerebellum. Neuroimaging studies and meta-analyses have yielded inconsistent results, with some reporting bilateral cerebellar activation (Imaizumi et al. 1997; Wildgruber et al. 2005; Ceravolo et al. 2021) and others only the left (Kotz et al. 2013) or right (Alba-Ferrara et al. 2011) activation during emotional prosody processing. However, a previous clinical study revealed an impairment of vocal emotion recognition in patients with left or right cerebellar lesions, particularly for neutral or negative prosody, but the former made fewer misattributions than the latter (Thomasson et al. 2021). The second NLSM analysis, which we carried out to further investigate the possible network underlying the specific performance of the RCBL group, revealed a network of regions located solely in the left parietal lobe, and more specifically, the left IPL, postC, and SMG. Interestingly, specific activation in the left SMG was observed during a linguistic prosody recognition task in which healthy participants had to judge whether or not a stimulus (intonated as a question or statement) had a different linguistic prosody from the previous one (1-back prosody task) (Kreitewolf et al. 2014). Activation in the cerebellum (contralateral to fronto-temporal activation) was also reported in this study. The authors suggested that the left SMG is associated with working memory strategies that involve covert rehearsal of pitch contours. This can be seen in the light of a previous study demonstrating that the right cerebellum plays a causal role in pitch processing (Lega et al. 2016). It is also in line with the results of research showing that misattributions by right cerebellar stroke patients can be explained by perceptual features such as pitch, loudness, and spectral aspect (Thomasson et al. 2021). However, our results also demonstrated deficits in emotion recognition in LCBL patients (for neutral prosody, with a trend toward a deficit for angry prosody), as had been observed in a previous clinical study (Thomasson et al. 2021). Thus, taken together, the present and previous findings suggest bilateral cerebellar involvement, both sensory and cognitive, in the processing of emotions conveyed by the human voice. This would be consistent with hypotheses formulated at the cortical level, which argue that both cortical hemispheres are essential to vocal emotional decoding and that it is the timescale for decoding the unfolding auditory information that drives lateralization. The right hemisphere would appear to be more closely related to low fluctuations that drive the ability to integrate large-scale information (e.g. pitch dynamics of voice), whereas the left hemisphere appears to be recruited more to discriminate short-scale information (e.g. phonemes) (Schirmer and Kotz 2006; Grandjean 2020). These results are in line with—and neatly complement—previous proposals regarding the functional specialization and integration of the cerebellum during emotion processing (Pierce and Péron 2020, 2022; Thomasson and Péron 2022). Sad prosody, for example, is characterized by lower intensity and variability in the fundamental frequency but with microstructural irregularities (i.e. short-term irregularities in fundamental frequency, intensity, and/or duration) (Juslin and Laukka 2003). A very fine temporal level of processing is therefore required to capture these microstructural irregularities, and the latter could therefore be underpinned by both the cerebellum—especially posterior and vermal regions—and the left cortical hemisphere. The basal ganglia (e.g. ventral portion of caudate and putamen, ventral external and internal globus pallidus, and medioventral subthalamic nucleus) could also participate to this processing by using the internal representation of the temporal structure to recruit and synchronize the activity of the cortical and subcortical structures required for this process. This also allows them to strengthen and refine units of previously established sequence representations (chunks) and even to build new units (Péron et al. 2013). These chunks may be modified by the cerebellum to minimize the prediction error of an internal model based on its ability to monitor the input and output and adjust the degree of its intervention according to the current context and feedback signals (Peterburs and Desmond 2016; Caligiore et al. 2019). In line with this assumption, a previous study comparing emotional prosody recognition performances in patients with Parkinson’s disease versus with cerebellar stroke suggested a crossed functional specialization between the basal ganglia and cerebellum according to the level of cognitive integration (Thomasson et al. 2022). Thus, these collaborative processes could be involved in both low-level and higher-level processing, be it in motor, cognitive, or emotional activities.

Overall, our data emphasize the importance of further studying patients’ emotional deficits by modeling them at the network level rather than trying to understand them solely on the basis of isolated regions. Any such studies will bring about crucial advances in knowledge about the functional specialization and integration of brain structures in emotions. They will also improve remediation in patients exhibiting emotional disorders based on possible compensatory mechanisms (Ruggiero et al. 2021).

### Limitations

The present study had several limitations that need to be acknowledged. First, although the main advantage of the discrimination index is that it provides information about a possible confusion or noisy emotional signal, its use results in a loss of information about patients’ possible identification biases. Second, NLSM is an innovative means of identifying the components of a network, but it does not yield any information about their hierarchical organization. Studies that measure the functional and effective connectivities during the execution of the task are needed to reveal the nature of functional alterations in emotional processing following cerebellar stroke—especially, as this method is blind to compensatory or maladaptive plastic changes within the damaged network. However, as mentioned by Saur et al. (2006), this method may be most valuable in the acute and subacute stages after stroke when a decrease in cerebral blood flow is often observed. This effect after cerebellar stroke is very well documented (Broich et al. 1987; Komaba et al. 2000), which further supports the use of this method in our study. Moreover, our analysis focused on the gray-matter portion of the lesion masks, as resting-state fMRI measurements are only informative for this tissue class (Logothetis et al. 2001; Buxton 2013). Thus, dysfunction between brain areas caused by damage in white-matter tracts was not considered in the present study. Finally, the absence of results for LCBL patients in our second VLSM analysis conducted with subgroups formed according to the lesion location does not necessarily mean that no neural networks are involved in sadness recognition in LCBL. A larger number of patients in this subgroup would perhaps have led to greater statistical power.

## Conclusion

This study revealed that the vocal emotion recognition in cerebellar stroke patients was linked to a cerebral cortical network involving left frontal, temporal, and parietal regions. Moreover, a more restricted network composed of regions located solely in the left parietal lobe was found to correlate with the performances of RCBL patients who displayed specific deficits for sadness recognition. These results suggest a specific functional specialization of both the cerebellum and the left cortical hemisphere in the processing of information on a fine temporal scale. Although NLSM is a powerful tool capable of delineating the architecture of functional networks underlying complex cognitive functions, additional studies are needed to further characterize the role of the cerebellum in emotional processes and identify its top-down and bottom-up influences.

## Supplementary Material

Supplementary_information_tgad002

## Data Availability

All data used for analysis are available upon reasonable request.
