# Supplementary material for: Dysfunctional cerebello-cerebral network associated with vocal emotion recognition impairments
Source: Cereb Cortex Commun. 2023 Jan 11;4(1):tgad002. doi: 10.1093/texcom/tgad002 (PMC9883615; doi:10.1093/texcom/tgad002)
Supplement: Supplementary_information_tgad002 [file supplementary_information_tgad002.docx]

**Supplementary information**

**Table S1. Mean score and 95% confidence interval (95% CI) for each emotion in the emotional prosody recognition task for patients (LCBL and RCBL subgroups) and HC**

| **^Patients (^*^n^* ^= 27)^** | | | | | | | | | | |
| --- | --- | --- | --- | --- | --- | --- | --- | --- | --- | --- |
|  | *^Happiness^* | | *^Fear^* | | *^Sadness^* | | *^Anger^* | | *^Neutral^* | |
|  | ^Mean^ | ^CI^_95%_ | ^Mean^ | ^CI^_95%_ | ^Mean^ | ^CI^_95%_ | ^Mean^ | ^CI^_95%_ | ^Mean^ | ^CI^_95%_ |
| ^Discrimination index^ | ^24.63^ | ^18.76, 30.49^ | ^30.15^ | ^24.28, 36.01^ | ^30.86^ | ^24.99, 36.73^ | ^42.49^ | ^36.62, 48.35^ | ^27.96 a^ | ^22.09, 33.82^ |
| **^LCBL subgroup (^*^n^* ^= 11)^** | | | | | | | | | | |
|  | *^Happiness^* | | *^Fear^* | | *^Sadness^* | | *^Anger^* | | *^Neutral^* | |
|  | ^Mean^ | ^CI^_95%_ | ^Mean^ | ^CI^_95%_ | ^Mean^ | ^CI^_95%_ | ^Mean^ | ^CI^_95%_ | ^Mean^ | ^CI^_95%_ |
| ^Discrimination index^ | ^24.27^ | ^15.89, 32.65^ | ^33.25^ | ^24.87, 41.63^ | ^36.88^ | ^28.50, 45.26^ | ^39.95^ | ^31.57, 48.33^ | ^26.05 a^ | ^17.67, 34.42^ |
| **^RCBL subgroup (^*^n^* ^= 16)^** | | | | | | | | | | |
|  | *^Happiness^* | | *^Fear^* | | *^Sadness^* | | *^Anger^* | | *^Neutral^* | |
|  | ^Mean^ | ^CI^_95%_ | ^Mean^ | ^CI^_95%_ | ^Mean^ | ^CI^_95%_ | ^Mean^ | ^CI^_95%_ | ^Mean^ | ^CI^_95%_ |
| ^Discrimination index^ | ^24.68^ | ^17.25, 32.10^ | ^27.82^ | ^20.39, 35.24^ | ^26.53 a^ | ^19.10, 33.95^ | ^44.04^ | ^36.61, 51.46^ | ^29.08 a^ | ^21.65, 36.50^ |
| **^HC (^*^n^* ^= 27)^** | | | | | | | | | | |
|  | *^Happiness^* | | *^Fear^* | | *^Sadness^* | | *^Anger^* | | *^Neutral^* | |
|  | ^Mean^ | ^CI^_95%_ | ^Mean^ | ^CI^_95%_ | ^Mean^ | ^CI^_95%_ | ^Mean^ | ^CI^_95%_ | ^Mean^ | ^CI^_95%_ |
| ^Discrimination index^ | ^28.94^ | ^23.12, 34.75^ | ^34.74^ | ^28.92, 40.55^ | ^36.07 b^ | ^30.26, 41.89^ | ^49.20^ | ^43.38, 55.02^ | ^38.88 b,d,c^ | ^33.07, 44.70^ |

*Note.* ^a^ significant if *p* value below 0.05 (Bonferroni corrected) in comparison with HC group; ^b^ significant if *p* value below 0.05 (Bonferroni corrected) in comparison with RCBL subgroup; ^c^ significant if *p* value below 0.05 (Bonferroni corrected) in comparison with LCBL subgroup; ^d^ significant if *p* value below 0.05 (Bonferroni corrected) in comparison with whole patient group.


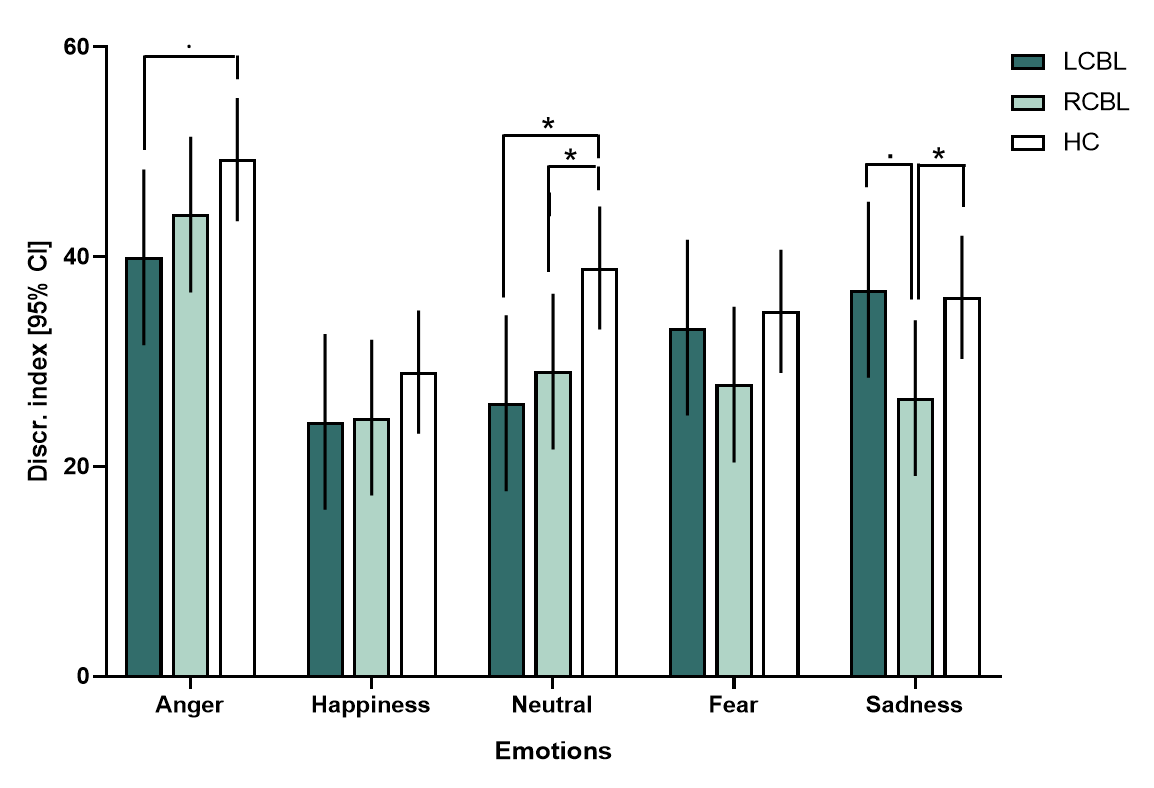


**Figure S1.** Mean discrimination index and 95% confidence interval (CI_95%_) for each emotion during the emotional prosody recognition task for LCBL, RCBL and HC groups.
